# From monocausality to systems thinking: a complementary and alternative conceptual approach for better understanding the development and prevention of sports injury

**DOI:** 10.1186/s40621-015-0064-1

**Published:** 2015-12-08

**Authors:** Adam Hulme, Caroline F. Finch

**Affiliations:** Australian Centre for Research into Injury in Sports and its Prevention (ACRISP), Federation University Australia, SMB Campus, PO Box 663, Ballarat, Victoria 3353 Australia

**Keywords:** Sports injury epidemiology, Socioecological frameworks, Systems thinking, Agent-Based Modelling, Applied Human Factors and Ergonomics

## Abstract

The science of sports injury control, including both its cause and prevention, has largely been informed by a biomedical and mechanistic model of health. Traditional scientific practice in sports injury research has routinely involved collapsing the broader socioecological landscape down in order to analyse individual-level determinants of injury - whether biomechanical and/or behavioural. This approach has made key gains for sports injury prevention research and should be further encouraged and allowed to evolve naturally. However, the public health, Applied Human Factors and Ergonomics, and injury epidemiological literature more broadly, has accepted the value of a socioecological paradigm for better understanding disease and injury processes, and sports injury research will fall further behind unless it does the same. A complementary and alternative conceptual approach towards injury control known as systems thinking that builds on socioecological science, both methodologically and analytically, is readily available and fast developing in other research areas. This review outlines the historical progression of causal concepts in the field of epidemiology over the course of the modern scientific era. From here, causal concepts in injury epidemiology, and models of aetiology as found in the context of sports injury research are presented. The paper finishes by proposing a new research agenda that considers the potential for a systems thinking approach to further enhance sports injury aetiological understanding. A complementary systems paradigm, however, will require that sports injury epidemiologists bring their knowledge and skillsets forwards in an attempt to use, adapt, and even refine existing systems-based approaches. Alongside the natural development of conventional scientific methodologies and analyses in sports injury research, progressing forwards to a systems paradigm is now required.

## Review

There is an Arabian proverb of which most are familiar. It speaks of a camel whose owner had overloaded it beyond what was a manageable weight; so much so, that it took only but a single piece of additional straw to bring the animal to its knees. The idiom, ‘the straw that broke the camel’s back’, now extends to scenarios where a certain number of precipitating factors combine to produce an undesirable outcome. In most instances, however, it is only the final event that is most noticeable to the person involved, and is routinely considered as the ‘unique cause’ of the effect in question (Rothman and Greenland 2005). The tendency for human beings to process events in this way, to otherwise implicate monocausality into their daily thinking, is simply a matter of habituation. Conversely, when deliberating over causality on a deeper level, or when attempting to formulate new scientific theories, one has to advance rudimentary conceptions of causality to that of complexity and multifactorialism (Rothman and Greenland 2005).

In the broader field of epidemiology, the science of causality, including ways in which to illustrate it, has been discussed at length (e.g. Greenland et al. 1999; Parascandola and Weed 2001; Broadbent 2013). Indeed, epidemiologists are not only motivated by the task of distinguishing simple correlation from actual causation, but also by the underlying and often times elusive and complex nature underpinning causal relationships (Glass et al. 2013). In other words, epidemiologists strive to identify both the risk factors for, and the causal mechanisms behind, the health effect in question. Closer to home, in the sports injury literature, there have been a number of examples that discuss or illustrate causality from a general prevention perspective (Meeuwisse 1994a, b; Gissane et al. 2001; Bahr and Holme 2003; Bahr and Krosshaug 2005; McIntosh 2005). Notwithstanding these examples, causal theory in sports injury epidemiology has entered into a period of inertia despite the availability of alternative conceptual causal approaches. Sports injury prevention research will not be able to make significant gains unless a number of important issues pertaining to causality are addressed.

The first section of this narrative review discusses the historical progression of causal concepts in the field of epidemiology more generally. This section, albeit summarised to include only a few noteworthy contributions, provides insight into why and how casual theory has evolved over time. From here, causal concepts in the broader field of injury epidemiology, and models of aetiology as found in the context of sports injury research are presented. The paper finishes with an overview of how a systems thinking approach has the potential to further enhance sports injury aetiological understanding.

## The roots of causal concepts in the modern scientific era

In 1880, at the Tenth International Congress of Medicine in Berlin, the German physician Robert Koch made a significant contribution to the field of microbiology and disease causality. Reflecting upon his research into the origin of Tuberculosis, Koch outlined three illustrious causal postulates (Rivers 1937): (i) that the parasite occurs in every case of the disease in question; (ii) that it occurs in no other disease as a fortuitous and non-pathogenic parasite, and; (iii) that after being fully isolated from the body and repeatedly grown in pure culture, it can cause the disease again. At the time, Koch’s postulates were designed to definitively establish whether a causal relationship existed between a single infectious agent and particular disease. Ironically, however, it was the limitations associated with these postulates that contributed to advancing aetiological understanding in this area (Fredricks and Relman 1996). Certainly, for some pathogenic bacterial species, the postulates were highly applicable. Yet, for other organisms, a clear violation of one or more of the postulates was found (Fredricks and Relman 1996).

The discovery of viruses in the early Twentieth century prompted a revision to Koch’s postulates. Rivers (1937) recognised that the monocausal exposure-disease framework was flawed, and warned of its continued application. Twenty years later, Huebner’s (1957) refinements to causal theory included, for the first time, the importance of epidemiological approaches alongside mere laboratory-based research. With the passing of yet another decade, the Five Realities of acute respiratory disease were formulated and supported the now accepted multicausal paradigm through recognising the importance of the individual’s biological constitution, and the influence of seasonal variation on the pathogenicity of certain agents (Evans 1967).

The further discovery of hundreds of new viruses transformed disease causality into a complex concept that included demographical, geographical and social layers. Accordingly, the historical progression of the science of infectious disease causality has been condensed into three distinct stages (Evans 1976): (i) the nature of the *agent* as a key focus (e.g. Koch’s postulates and monocausality); (ii) consideration to the *environment* in which the disease occurred (e.g. refinements from Huebner and Rivers) (Huebner 1957; Rivers 1937); and (iii), recognition of how the characteristics of the *host* influences the pathophysiology of disease (e.g. Evan’s Five Realities) (Evans 1967).

### The evolution of causal thinking in epidemiology

Over the course of the mid-late 1900s, the provision of healthcare services, improved community sanitation and hygiene, and scientific discoveries including the development of vaccinations contributed to a declining incidence of infectious diseases (Baum 2011). Paradoxically, technological advancements and obesogenic environments gave rise to a range of new health issues. The epidemiological teaching resources that emerged around the 1950s embraced a new research agenda, and alongside infectious disease, were now concerned with the development and prevention of non-communicable chronic health conditions (Krieger 1994). The single agent germ theory was completely displaced by models of disease aetiology that directly assimilated, or took advantage of, the underpinning principles associated with the Agent, Host, and Environment triad. Standout examples include the Web of Causation (MacMahon et al. 1960), Hill’s (1965) nine considerations for inferring causation, and Rothman’s (1976; 2005) Theoretical Sufficient-Component Cause Model; which, was based on earlier work by distinguished philosophers of science (Mackie 1965; Lyon 1967).

## Causal concepts in injury epidemiology

Whether it be improvised footwear to protect against the elements, or engineered clothing and equipment worn during warfare, injury prevention interventions have continued to evolve since the earliest known records (Rivara 2001). Aside from a number of early lessons, it took until the mid-late Twentieth century before the true application of epidemiological techniques for better understanding injury control were applied (Rivara 2001). If it were not for the causal concepts that has previously been established in the infectious and chronic disease literature, injury epidemiology might have set out on an altogether different trajectory (Robertson 2007). Notable early concepts that were applied to injury control included the Domino Theory of Accident Causation (Heinrich 1931), De Dehaven’s (1942) biomechanical theories of energy exchange and force distribution, and the self-involved experiments of Stapp (1957). A number of influential visionaries prophetically elaborated on these robust theoretical foundations, and so injury control was established as a legitimate scientific discipline.

In his paper *‘The Epidemiology of Accidents’*, Gordon (1949) illustrated a similar pattern of mortality between an outbreak of typhoid fever amongst a troupe of circus performers and that of a nightclub fire. The analogy of these two distinct scenarios, aside from the literal graphical representation of the sharp and initial aggregation of cases, was reflected in his commentary (Gordon 1949; p.515):

*“Specifically directed prevention based on an understanding of cause has long guided the attack on communicable and other diseases…the biologic principles that govern disease as a community problem are interpreted as holding equally well for injuries. A pattern for epidemiologic analysis is presented [Agent, Host, and Environment], as a means for a better understanding of accidents”.*

Gordon (1949) believed that, like disease, injuries were caused by particular epidemiologic episodes, such as seasonal change, demographic characteristics and an individual’s susceptibility. Just over a decade later, an experimental psychologist proposed that injuries were caused by the transfer of energy (Gibson 1961). The theory of energy exposures exceeding an organism’s physiological injury threshold remains foundational to the science of injury control.

A breakthrough in injury research arrived with the release of *‘Accident Research: Methods and Approaches’* (Haddon et al. 1964). Haddon et al. (1964) had produced the ultimate anthological resource which established injury research as an important scientific discipline (Li and Baker 2014). The theories and methods presented in their definitive text were the catalyst for many more important publications that followed, including numerous reports by the US-based National Research Council and the National Highway Traffic Safety Administration (Rivara 2001). Like Gordon (1949) before him, one of the greatest contributions to injury research by Haddon (1970, 1980) was his recognition of the Agent, Host and Environment triad. Haddon’s (1970, 1980) efforts to coordinate three distinct injury prevention phases (i.e. pre-event, event, post-event) with the Epidemiological Triad resulted in the now famous Haddon Matrix for injury prevention interventions. The Haddon Matrix is widely used to conceptualise the candidate risk factors, temporality, and the mechanisms of injury, and has been applied in a number of different injury contexts (Scott-Parker and Morang MacKay 2015). The addition of a third dimension to Haddon’s Matrix by Runyan (1998) introduced value criteria to enhance the efficacy and effectiveness of injury prevention interventions. Runyan’s (1998) suggested criteria included: (i) effectiveness; (ii) cost; (iii) freedom; (iv) equity; (v) stigmatization; (vi) preferences, and; (vii) feasibility.

## Causal concepts in sports injury epidemiology

Contemporary models of sports injury aetiology have broadly visualised how a multitude of risk factors predispose and subsequently leave athletes susceptible to sustaining injury. These models have developed incrementally over time, being grounded in the broader causal concepts that have been outlined thus far.

**Fig. 1:**
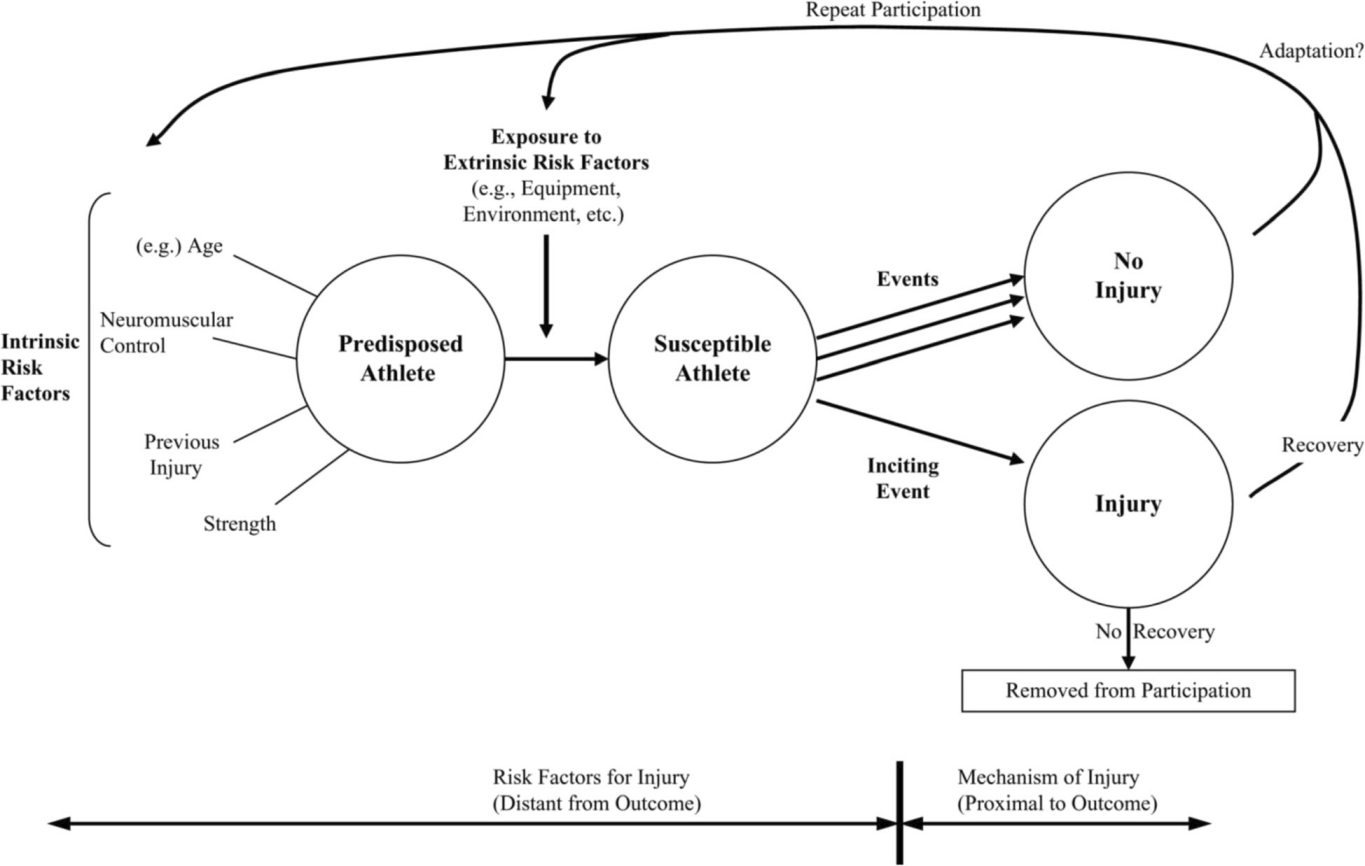
A dynamic, recursive model of etiology in sport injury (Meeuwisse et al. 2007)

Meeuwisse (1994a) was one of the first sports medicine researchers to discuss the importance of accurately assessing causation in sports injury research. In particular, two early articles outlined key principles relating to the assessment of risk factors, and elucidated why a multifactorial approach to understanding sports injury risk was needed (Meeuwisse 1994a, b). Inspired by causal concepts in the disease literature, Meeuwisse (1994a) created his new multifactorial model of athletic injury aetiology. The model included the relationship between intrinsic (e.g. maturational stage, somatotype, biomechanics, conditioning) and extrinsic (e.g. weather, footwear, terrain, competitive rules) risk factors and sports injury. According to the model, any given athlete has a unique predisposition for injury based on their own intrinsic set of risk factors, and further external risk factors acting ‘from outside’ render the athlete susceptible to injury. The multifactorial model was revised just over a decade later, prompted in part by the presentation of a new operational cyclical model by Gissane et al. (2001), alongside later suggestions (Bahr and Holme 2003; Bahr and Krosshaug 2005). The updated model effectively advanced the initial linear paradigm of injury causality to a dynamic model in which a given athlete’s susceptibility to injury could continually change according to many adaptations or maladaptations that occur with continued sports participation (Meeuwisse et al. 2007) (Fig. 1).

A biomechanical perspective on sports injury causality illustrated a number of considerations that added complexity to sports injury causality (McIntosh 2005). McIntosh (2005) drew upon his own research, and rationalised that the use of protective headgear might not affect sports injury risk, for better or worse, if that particular intervention were to modify the behaviour and attitudes of its user. The model visualised how personality, level of competitiveness and exposure to coaching practices interplay with environmental and biomechanical properties to influence injury risk. Ultimately, McIntosh (2005) explained how injury prevention programs might not work to their full capacity if physical loads are reduced through intervention, yet an increase in kinetic energy exchange and higher forces are encouraged through the actions and desires of coaches and athletes. Hagel and Meeuwisse (2004) similarly dedicated an earlier paper to the notion of risk compensation in the sports injury context. They argued that, despite the best intentions of researchers’ to introduce sports injury countermeasures, interventions might not always have the desired effect. Their conclusion emphasised the importance of conducting injury prevention studies for determining whether countermeasures are efficacious through evaluating their net benefit (Hagel and Meeuwisse 2004).

## Proposing a complementary research agenda for sports injury aetiological research

Existing sports injury prevention frameworks have been valuable for outlining and facilitating the overall research process. For instance, stage two associated with both the Sequence of Prevention model (van Mechelen et al. 1992) and the Translating Research into Injury Prevention Practice (TRIPP) framework (Finch 2006) specifies that the implementation and evaluation of injury prevention interventions cannot occur until risk factors and mechanisms of injury have been firmly established. Accordingly, aetiological research requires a multidisciplinary approach, including not only biomechanical and clinical studies, but also investigations with a focus on behavioural and motivational factors (Finch 2006). Despite this, very few original studies in the unintentional injury and sports injury literature have used behavioural and social science theories in order to facilitate the uptake and maintenance of injury prevention interventions (Trifiletti et al. 2005; McGlashan and Finch 2010). This is concerning given that injury research, whether focused on aetiology or prevention, has to occasionally reach above and beyond not only the biomedical and clinical sciences, *but also* the behavioural and motivational levels to truly make a difference (Allegrante et al. 2010). In other words, incorporating injury determinants as they relate to policy development and legislation are also crucial for prevention purposes, yet they too have only featured on a very limited basis in sports injury research.

In one of very few examples, Cameron et al. (1994) explained that before regulations specified that bicycle helmet use was mandatory, the overall uptake of this injury prevention intervention was less than adequate. If people do not know, appreciate, or consider that particular injury countermeasures are necessary for enhancing their personal safety, there will be little incentive to use them. Accordingly, to increase the effectiveness of an intervention to reduce severe eye injuries amongst squash players, Eime et al. (2005) collaborated with the Victorian Squash Federation, leading eyewear manufacturing companies and sports venue managers. This was alongside behavioural and motivational strategies to ensure both the uptake and efficacy of the program was successful (Eime et al. 2004).

More recently, Finch and Donaldson (2010) developed a novel extension to the RE-AIM (Reach; Effectiveness; Adoption; Implementation; Maintenance) framework, through the Sports Settings Matrix to identify the multiple levels of the sports delivery setting (e.g. national level through to a club, team and individual level that impact on injury prevention). The authors’ stressed that the attitudes and knowledge towards injury prevention interventions need addressing, but equally, the setting, culture, and infrastructural support networks in which programs are to be delivered are also essential considerations for the success of initiatives. For injury prevention interventions to have the best chance of working, practice-based research that aims to measure the contextual determinants of program effectiveness is required to translate efficacy into effectiveness; but alone, this is not enough. Even prior to implementation, it is imperative to reconcile differing perceptions of injury causation (Hanson et al. 2012).

### The current state of sports injury aetiological research

Contemporary models of sports injury aetiology have been influenced by a doctrine of scientific objectivity and engineered under a biomedical construct. This means that injury mechanisms have primarily been understood from a biophysiological and biomechanical perspective. Despite being useful for calibrating research priorities and enhancing injury prevention efforts, such models have always directed attention to the individual athlete (i.e. age, gender, strength, neuromuscular control, equipment, training surface etc.) (Meeuwisse 1994a; Gissane et al. 2001; McIntosh 2005; Meeuwisse et al. 2007). This promotes a view that the science of sports injury control is best characterised by reducing the injury mechanism down to a level that only educational, behavioural and medically-oriented interventions can address. If not called into question, a biomedical and objectivist epistemic tradition will continue to lead sports injury researchers to believe that athletes are ‘free’ agents who can always ‘choose’ their own behaviours. What is now required is the introduction of a complementary and alternative conceptual approach for better understanding the development and prevention of sports injury. Revisiting the ten ecological principles (Haddon 1970) and re-examining the Injury Iceberg (Hanson et al. 2005) represents the first step in being able to show that it is possible to preserve the traditional approach in sports injury research, yet simultaneously, extend the horizon beyond it.

### Forwards to a systems paradigm

Over the latter half of the Twentieth century, the field of public health blossomed into a multidisciplinary science (Rogers 1960). The limitations associated with routinely targeting interventions at individual-level, health-related determinants were recognised (Rose 1985; Graham 2004). The controversial ‘*The role of medicine. Dream, Mirage or nemesis*’ claimed that the primary reasons for improvements in health-related outcomes in the developed world, at least post Eighteenth century, were nutritional, environmental and behaviourally-related (McKeown 1979). With increasing awareness that the process of scientific reductionism was not the sole answer to many public health issues, early government policies (Lalonde 1974), associated literature (Blum 1974; Dever 1976), and pivotal comprehensive global agendas by the World Health Organisation (1986) recalibrated focus upstream to a political and societal-level (Graham 2004). Around the same time, calls for a greater emphasis to be placed on social science theory (Cassel 1964, 1976) and social reformation strategies to address socioeconomic inequalities (Wing 1984, 1988) started to catalyse some of the more recognised ecological models of health (e.g. Dahlgren and Whitehead 1991; Green and Kreuter 1999; VanLeeuwen et al. 1999).

A reorientation of focus to upstream health-related determinants nurtured a quiet tension and scientific divide with regard to how disease pathogenesis and pathophysiology could best be investigated and understood. One school of scientists preferred to reduce disease down to a molecular level and study its pathogenic mechanisms, especially given technological advances in the fields of biology and genetics (Vandenbroucke 1988). Vandenbroucke (1988) drew a comparison between the Nineteenth century’s miasmic theory and the modern day environmentalist movement striving for social change. Conversely, another school of scientists believed that historical, social, and geographical factors had been, and still were, equally responsible for the aetiology of many diseases alongside the specific-agent position (Loomis and Wing 1990). Loomis and Wing (1990) identified the similarity between Vandenbroucke’s (1988) molecularised epidemiology and the previous century’s germ theory.

In *‘The Limits of Epidemiology’*, Wing (1994) claimed that the field of epidemiology was vulnerable to being labelled as a ‘basic science’ if practitioners were to continue viewing exposure-disease relationships as self-contained, homogenous and universal phenomena. In other words, generalised assumptions and inferences derived via experimental and observational study designs need to reflect the social, political, and economic dimensions to which exposure(s) are influenced (Wing 1994). Consequently, in a series of papers, Susser and Susser (1996a, b) and Susser (1998) argued that the field of epidemiology required a theoretical shift to encourage the emergence of a new scientific paradigm titled ‘eco-epidemiology’. The brilliance of this work, though, was not necessarily with a proposed eco-epidemiological paradigm, but the ability to outwardly project into the future (Susser and Susser 1996b; p.676):

*“…one must also take heed of another emergent paradigm. Information systems combined with systems analyses might well lead into a systems paradigm, with its own attractions for mathematically minded epidemiologists…”.*

**Fig. 2:**
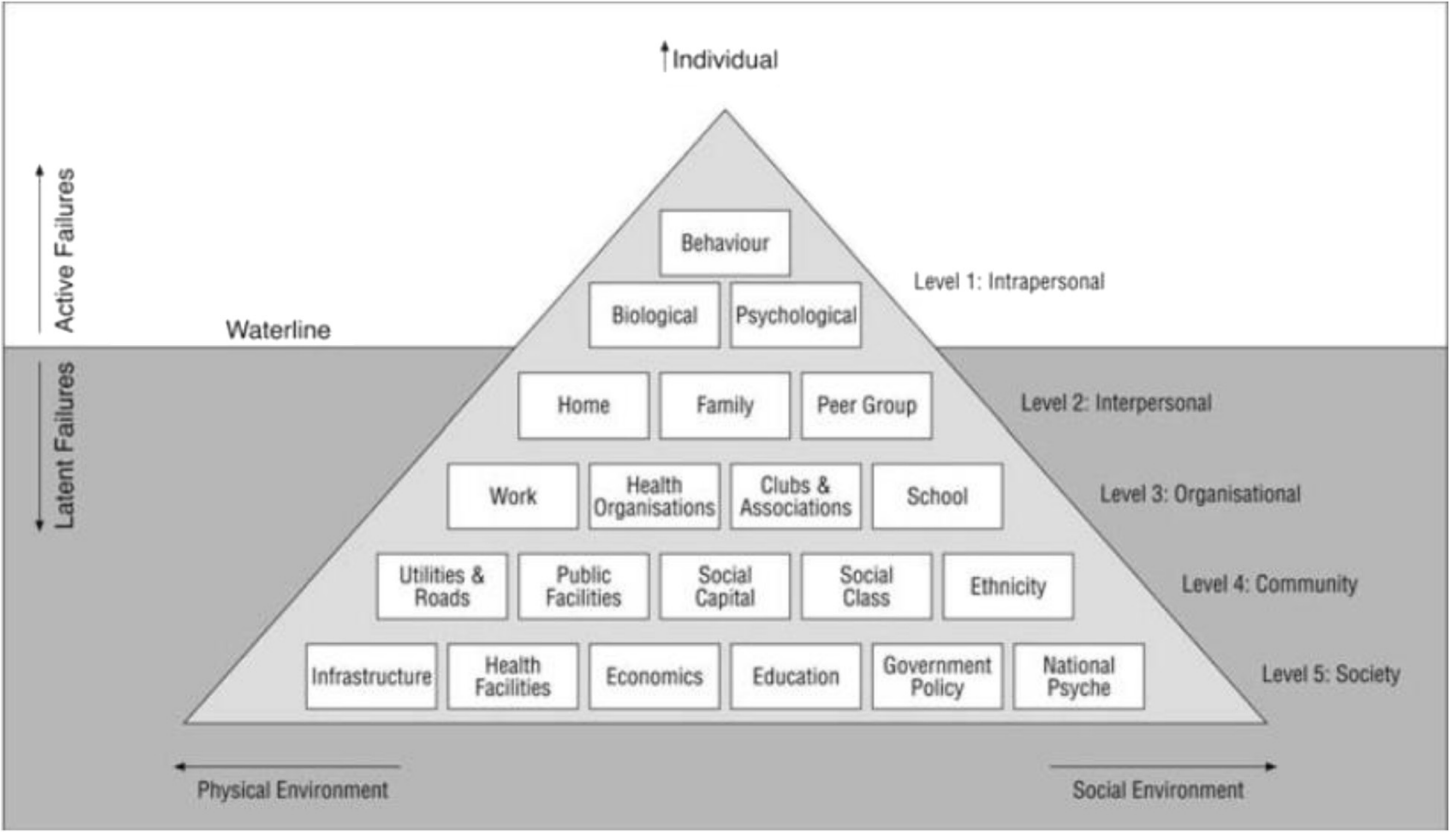
The Injury Iceberg (Hanson et al. 2005)

With recognition for Haddon’s (1970) early concept of ecological injury prevention, and Green and Kreuter’s (1999) ecological approach in the context of health promotion, Hanson et al. (2005) presented their metaphorical iceberg of injury prevention for the application of community safety interventions. The model visualised that above the water’s surface and within the iceberg’s tip lies a single level containing: (i) intrapersonal factors (e.g. behaviour, biology, psychology). But below the waterline in the socioecological depths were an additional four levels. These were: (ii) interpersonal (e.g. home, family); (iii) organisational (e.g. occupation, heath organisations); (iv) community (e.g. social class, public facilities), and; (v) society (e.g. infrastructure, government policy). Both Haddon (1970) and Hanson et al. (2005) identified that the aetiology and prevention of injury, like disease, is grounded in an intrinsically ecological concept, and the individual is merely the salient ‘tip’ of the iceberg (Fig. 2).

In the case of the Injury Iceberg, a socioecological perspective towards injury control has many benefits, and draws attention to: (i) the importance of ergonomic and environmental design; (ii) the sustainability and allocation of resources (e.g. personnel availability to financial budgeting); (iii) the value of community engagement and empowerment, and; (iv) how multiple countermeasures and interventions at different levels can maximise the ability to attenuate risk and prevent injury (Hanson et al. 2005; Allegrante et al. 2010). On the other hand, socioecological models are, first and foremost, only conceptual frameworks in which to challenge the biomedical paradigm of individualism which originated out of the ‘medical model’ of both disease and injury (Eime et al. 2004, 2005). For instance, any given socioecological model does not identify discrete factors, nor does it attempt to substantiate the strength and temporality of causal effects *across* its entire framework. In a similar manner, Hill’s (1965) considerations for causation and Rothman’s (1976; 2005) model, despite having had a positive impact on contemporary epidemiological issues (e.g. Potischman and Weed 1999; Grant 2009; Ronksley et al. 2011), have also been regarded as ‘heuristics’ that are limited in their scope and application (Koopman and Lynch 1999; Phillips and Goodman 2004, 2006; Marshall and Galea 2014). Notwithstanding the promising evolution of multicausal theory in epidemiology, many important public health issues stand resilient in spite of the best intentions to design and implement suitable interventions (Marshall and Galea 2014). Another conceptual approach known as ‘systems thinking’, which builds on the strong theoretical foundation that is offered by socioecological models, has potential and should be considered for better understanding the development and prevention of sports injury.

#### Thinking in ‘systems’

Systems thinking is a unique science that partly emerged out of General Systems Theory (Bertalanffy 1969), and has been further refined by academics from the fields of engineering and organisational safety (e.g. Checkland 1981; Ackoff 1971) alongside scientists located at the Massachusetts Institute of Technology (Senge 1990). Systems thinking shares the multifaceted framework that is offered by socioecological models of health (Dahlgren and Whitehead 1991; Green and Kreuter 1999; VanLeeuwen et al. 1999; Hanson et al. 2005), but elaborates with its own theory and principles. In other words, socioecological and systems thinking approaches are conceptually synonymous, but particular systems thinking techniques offer methodological and analytical rigour to an already primed ecological framework. A succinct definition of systems thinking has been provided by Trochim et al. (2006; p.593):

*“Systems thinking is a general conceptual orientation concerned with the interrelationships between parts and their relationships to a functioning whole, often understood within the context of an even greater whole. It is ancient in origin and familiar to us all, but it is also something very modern”.*

### System thinking theory and principles

A number of systems thinking principles are well recognised (Sterman 2006; Diez Roux 2007; Dekker 2011): (i) complexity in the system arises from multiple webs, relationships, and interactions between a large number of heterogeneous factors; (ii) the knowledge associated with a given actor, agent or factor in the system is limited and localised to its respective sub-system or level; (iii) history plays an important role in the system, and past events explain present and future behaviour; (iv) interactions in the system can include non-linear self-reinforcing and self-correcting feedback loops (i.e. reciprocity), which might produce an emergent effect (i.e. small initial events can reverberate exponentially and produce a disproportionately larger consequence in time, otherwise known as ‘sensitivity on initial conditions’); (v) complex systems are homeostatic: they persist, adapt, and are continually in flux to enable reconfiguration in response to internal or external influence and change; (vi) systems are counterintuitive, and aetiological processes can be vastly distant in time and space, and; (vii) systems can be resistant to obvious solutions (i.e. seemingly reasonable strategies can actually worsen the issue). The corollary of these characteristics is that adverse events, including injury, are emergent properties that arise from the many decisions, actions and interactions between actors and agents across the entire system.

### Principles in practice

Systems thinking principles violate the overall premise associated with the ‘chain-of-causality model’ (Leveson 2011). For instance, simply working backwards from the injurious outcome, whilst pinpointing particular failures interspersed by human error, is a process divorced from systems concepts such as nonlinearity and emergence. In a similar way, epidemiologists generally prioritise the study of proximal downstream causal effects (Glass et al. 2013). Certainly, it is more feasible to conduct observational studies and randomised controlled trials with cohorts of individuals, than it is to examine the nature of upstream influences across the broader social structure (i.e. the behaviour of powerful corporate entities, tax systems, and political processes) (Hernán 2015). This point is no better illustrated than by current models of sports injury aetiology which have primarily been concerned with the individual athlete and their immediate environment. The revised model of athletic injury aetiology (Meeuwisse et al. 2007), for example, represents a sound attempt at advancing the initial static and linear paradigm, but systemic and interpersonal determinants are not featured. According to systems theory, any given system is characterised by continual adaptation and change involving multiple sub-systems. These sub-systems are further comprised of many interconnected components that are fundamentally different, including non-biological elements (e.g. amenities, products), along with individuals, communities, organisations, regulatory agencies and political bodies. Ultimately, injury is the result of the many *complex interrelated processes* that need to be understood, and not the events and conditions in the system *per se* that produce emergent behaviour (Leveson 2011).

The application of system-based principles also have a number of analytical implications in terms of conventional epidemiological approaches. For example, given that it is necessary to study a system as a whole instead of isolating relationships between individual factors, the underlying assumptions that are commonly used in traditional statistical modelling are divorced from systems theory (Ip et al. 2013). This does not mean that systems thinking dismisses or acts as a substitute for scientific reductionism or linear modelling. Rather, system-driven approaches are viewed as supplementary to reductionist approaches, and can even include data derived via traditional statistical methods (Trochim et al. 2006). In response to the promulgation of an ecological understanding of health-related processes, more sophisticated analytical techniques are available, such as multilevel analyses and random effects models (Luke and Stamatakis 2012; Galea and Ahern 2006). Despite being able to adjust for potential confounding, a fundamental limitation associated with all regression-based analyses lies with their inability to account for system-wide phenomena, such as self-reinforcing and self-correcting feedback mechanisms or causal effects that are time-distant from the outcome (i.e. the use of longitudinal snapshot data at predefined intervals) (Galea et al. 2010). Illustrative models such as Directed Acyclic Graphs and Structural Equation Modelling (e.g. path analysis) are used for different ends, and have even featured in sports injury research (e.g. Shrier and Platt 2008). However, these types of diagraphs are mostly concerned with the visualisation of traditional statistical-related subject matter at a single level (e.g. adjusted effect estimates and directed dependencies) (Pearl 1995; Greenland et al. 1999; Shipley 2002; Greenland 2003; Olobatuyi 2006; VanderWeele and Robins 2007). Effectively, a systems approach attempts to understand the underlying processes along with the *overall functioning* of a system in relation to its principles, rather than to identify individual causal effects between isolated parameter estimates (Diez Roux 2007).

### A brief overview of available system-based methods

Although not formally recognised as such in the literature, there are two main systems-related fields. At one end of the systems thinking continuum lies computational system science methods which includes three prominent techniques: Agent Based Modelling (ABM), System Dynamics, and Network Analysis (Bonabeau 2002; Trochim et al. 2006; Marshall and Galea 2014). Both ABM and System Dynamics are computer-based simulations that have the ability to produce emergent behaviour after equations and rules have been assigned to individual elements in the system (Resnicow and Page 2008; Galea et al. 2010; Luke and Stamatakis 2012) (i.e. predict the potential spread of global infectious pandemics and patterns of climate change). These methods, however, have also had epidemiological applications to chronic disease (Ness et al. 2007), Human Immunodeficiency Virus transmission and prevention (Aral et al. 2010; Marshall et al. 2012), substance-abuse (Moore et al. 2009; Gordon et al. 2006), physical inactivity (Yang et al. 2011), and dietary practices (Auchincloss et al. 2013). In terms of injury, System Dynamic modelling has been discussed as a way to strengthen the understanding of upstream activities in order to identify key political leverage points for injury prevention purposes (Ferencik and Minyard 2011).

The other end of the systems thinking armamentarium belongs to the science of Applied Human Factors and Ergonomics (AHFE), which historically, has been concerned with the detailed analyses of accidents. The AHFE literature also contains three prominent systems-based methods (Salmon et al. 2012): Rasmussen’s (1997) Risk Management Framework, Reason’s (1997) Swiss Cheese model (Salmon et al. 2010), and Leveson’s (2004) Systems Theoretic Accident Modelling and Processes model. Application of these three AHFE systems methodologies has been dependent on the skill and experience of the systems analysts who have used them. Historical data, witness reports, expert consensus, and a range of other qualitative methods of inquiry are required to facilitate the identification of system failures associated with accidents and injury. This subjectivity could be regarded as an inherent limitation associated with these methods and models. Nevertheless, AHFE systems-based methods have been successfully used for accident analysis and injury control purposes in a number of contexts, including the firearm (Jenkins et al. 2010), industrial (Goode et al. 2014), rail (Read et al. 2013), outdoor activity (Salmon et al. 2014), and road safety (Scott-Parker et al. 2015) systems. The successful operationalisation of AHFE systems-based methods demonstrates that these approaches are viable, practical, and highly versatile.

### A case in point from the sports injury literature

The potential value of systems thinking principles can be illustrated in the context of sports injury by using the activity of distance running as an example. This particular exercise modality, whether for recreational or competitive ends, has been chosen given its popularity, accessibility, and the plethora of health-related benefits associated with it. The term ‘the distance running system’ will exemplify this scenario based on research from another context (Scott-Parker et al. 2015).

The distance running system in which a runner resides is comprised of many levels, including but not limited to: (i) equipment and the physical training environment; (ii) the runner themselves; (iii) wider social networks including other runners; (iv) occupational habits and lifestyle practices; (v) fitness trainers and coaches; (vi) running and fitness clubs and associated policies; (vii) community healthcare services; (viii) athletics associations and official governing bodies, and; (ix) the wider political and regulatory environment. Certain outputs in the distance running system, such as injury, result from the synergistic interaction between its many various heterogeneous elements. Consistent with contemporary models of sports injury aetiology (Meeuwisse et al. 2007), the most utilised epidemiological approach has been to collapse the distance running system down, and reduce injury mechanisms to the biomechanical and behavioural levels only (van Gent et al. 2007; Nielsen et al. 2012; Saragiotto et al. 2014). From there, it has been possible to examine particular causal effects of interest (e.g. Boldt et al. 2013; Bredeweg et al. 2013; Rodrigues et al. 2013; Nielsen et al. 2014), such as isolating the association between isokinetic strength variables and injury using traditional statistical modelling (e.g. Messier et al. 1995). On the other hand, reassembling the distance running system after identifying statistically significant variables (on the tacit assumption that the whole cannot be greater or less than the sum of its parts), now requires supplementation with a systems approach. This will involve traversing ‘up and out’ of the system to also identify and examine the contribution of indirect influences and systemic processes as they relate to running injury development. This includes, for example, the marketing, distribution and uptake of running footwear, the design of built environments, social expectations and norms, emerging technologies and the role of ‘e-health’, athletic policies, and the influence of private industry and healthcare services.

In reconciling systems concepts with epidemiology, Pearce and Merletti (2006) argue that the health of a population can be viewed as a complex adaptive system. By definition, this premise can be extended to athletic populations, including distance runners. But as Diez Roux (2007) has reasonably asked, what would a systems perspective actually *look like* in practice? The answer to this question is dependent on which systems-related field and method is adopted. Computational system science techniques and AHFE methods both show great promise for a variety of topics, but the former are inherently quantitative and the latter qualitative. It can be said with certainty, however, that both ends of the systems thinking continuum necessitate a team of multidisciplinary practitioners, each with unique skillsets and knowledge regarding how to operationalise a particular methodology (Ferencik and Minyard 2011). In order to answer questions about complex causal phenomena, epidemiologists are encouraged to find the ‘middle ground’ between traditional epidemiological inquiry, and the abstract mental models found in the social sciences (Marshall and Galea 2014; Hernán 2015). Even though systems thinking is an appropriate starting point in which to reconcile data with theory, it still remains to be widely accepted across a number of scientific disciplines. This is because system-based methods are still in a stage of maturation and refinement. Currently, it is not possible to produce a numerically precise systems-based model that simultaneously preserves the face validity underpinning the nature of reality (Ip et al. 2013). This delicate balance between statistical precision and ecological realism, however, might be viewed as a welcome trade-off for sports injury prevention research. Therefore, we contend that the future study of causality in sports injury research lies with a mutually inclusive answer: continue to utilise traditional epidemiological approaches, but also embrace the possibilities associated with a systems thinking approach.

## Conclusion

Reflecting back on the historical context in which causal concepts in epidemiology have been formulated is important for establishing scientific progress, and presents the opportunity to inform future perspectives. The journey from the theory of monocausality in the late Nineteenth century to multifactorialism in the modern scientific era is only the beginning. Bar a few exceptions to the general rule, the main focus of sports injury aetiological research to date has been on risk factor identification at the individual component cause level. Some have argued that the next step for sports injury research is to further embrace an ecological perspective that supplements the biomedical tradition – both in terms of aetiology and the implementation of injury prevention interventions. However, rapid developments in the broader field of public health and Applied Human Factors and Ergonomics, are fast moving beyond the socioecological era. In fact, recognition for the potential of systems thinking methodologies and analyses has already gained traction in other injury contexts. The advancement of sports injury prevention research will require that epidemiologists bring their knowledge and skillsets forwards in an attempt to use, adapt, and even refine existing systems-based approaches. Alongside the natural development of conventional scientific methodologies and analyses in sports injury research, moving forwards to a complementary systems paradigm is now required.

## Abbreviations

ABM: Agent Based Modelling
AHFE: Applied Human Factors and Ergonomics
RE-AIM: Reach, Effectiveness, Adoption, Implementation, Maintenance

## References

[CR1] Ackoff RL (1971). Towards a system of systems concepts. Manag Sci.

[CR2] Allegrante JP, Hanson DW, Sleet DA, Marks R (2010). Ecological approaches to the prevention of unintentional injuries. Ital J Public Health.

[CR3] Aral SO, Leichliter JS, Blanchard JF (2010). Overview: the role of emergent properties of complex systems in the epidemiology and prevention of sexually transmitted infections including HIV infection. Sex Transm Infect.

[CR4] Auchincloss AH, Riolo RL, Brown DG, Cook J, Roux Diez AV (2013). An agent-based model of income inequalities in diet in the context of residential segregation. Am J Prev Med.

[CR5] Bahr R, Holme I (2003). Risk factors for sports injuries - a methodological approach. Br J Sports Med.

[CR6] Bahr R, Krosshaug T (2005). Understanding injury mechanisms: a key component of preventing injuries in sport. Br J Sports Med.

[CR7] Baum F (2011). The new public health.

[CR8] Bertalanffy L (1969). General system theory: foundations, development, application.

[CR9] Blum HL (1974). Planning for health: developmental application of social change theory.

[CR10] Boldt AR, Willson JD, Barrios JA, Kernozek TW (2013). Effects of medially wedged foot orthoses on knee and hip joint running mechanics in females with and without Patellofemoral Pain Syndrome. J Appl Biomech.

[CR11] Bonabeau E (2002). Agent-based modeling: methods and techniques for stimulating human systems. Proc Natl Acad Sci U S A.

[CR12] Bredeweg SW, Kluitenberg B, Bessem B, Buist I (2013). Differences in kinetic variables between injured and noninjured novice runners: a prospective cohort study. J Sci Med Sport.

[CR13] Broadbent A (2013). The philosophy of epidemiology.

[CR14] Cameron MH, Vulcan AP, Finch CF, Newstead SV (1994). Mandatory bicycle helmet use following a decade of helmet promotion in Victoria, Australia – an evaluation. Accid Anal Prev.

[CR15] Cassel J (1964). Social science theory as a source of hypotheses in epidemiological research. Am J Public Health Nations Health.

[CR16] Cassel J (1976). The contribution of the social environement to host resistance. Am J Epidemiol.

[CR17] Checkland P (1981). Systems thinking, systems practice.

[CR18] Dahlgren G, Whitehead M (1991). Policies and strategies to promote equity in health.

[CR19] De Haven H (1942). Mechanical analysis of survival in falls from heights of fifty to one hundred and fifty feet. War Med.

[CR20] Dekker S (2011). Drift into failure.

[CR21] Dever AGE (1976). An epidemiological model for health policy analysis. Soc Indic Res.

[CR22] Diez Roux AV (2007). Intergrating social and biologic factors in health research: a systems view. Ann Epidemiol.

[CR23] Eime R, Owen N, Finch CF (2004). Protective eyewear promotion: Applying principles of behaviour change in the design of a squash injury prevention programme. Sports Med.

[CR24] Eime R, Finch C, Wolfe R, Owen N, McCarty C (2005). The effectiveness of a squash eyewear promotion strategy. Br J Sports Med.

[CR25] Evans AS (1967). Clinical syndromes in adults caused by respiratory infection. Med Clin North Am.

[CR26] Evans AS (1976). Causation and disease: the Henle-Koch postulates revisited. Yale J Biol Med.

[CR27] Ferencik R, Minyard K (2011). Systems thinking and injury prevention: an innovative model for informing state and local policies. West J Emerg Med.

[CR28] Finch CF (2006). A new framework for research leading to sports injury prevention. J Sci Med Sport.

[CR29] Finch CF, Donaldson A (2010). A sports setting matrix for understanding the implementation context for community sport. Br J Sports Med.

[CR30] Fredricks DN, Relman DA (1996). Sequence-based identification of microbial pathogens: a reconsideration of Koch’s postulates. Clin Microbiol Rev.

[CR31] Galea S, Ahern J (2006). Invited commentary: considerations about specificity of associations, causal pathways, and heterogeneity in multilevel thinking. Am J Epidemiol.

[CR32] Galea S, Riddle M, Kaplan GA (2010). Causal thinking and complex system approaches in epidemiology. Int J Epidemiol.

[CR33] Gibson JJ, Jacobs HH (1961). The contribution of experimental psychology to the formulation of the problem of safety: a brief for basic research. Behavioral approaches to accident research.

[CR34] Gissane C, White J, Kerr K, Jennings D (2001). An operational model to investigate contact sports injuries. Med Sci Sports Exerc.

[CR35] Glass TA, Goodman SN, Hernán MA, Samet JM (2013). Causal inference in public health. Annu Rev Public Health.

[CR36] Goode N, Salmon PM, Lenné MG, Hillard P (2014). Systems thinking applied to safety during manual handling tasks in the transport and storage industry. Accid Anal Prev.

[CR37] Gordon JE (1949). The epidemiology of accidents. Am J Public Health Nations Health.

[CR38] Gordon DM, Mezic J, Gruenwald PJ (2006). Agent-based modelling of drinking behavior: a preliminary model and potential applications to theory and practice. Am J Public Health.

[CR39] Graham H (2004). Social determinants and the unequal distribution: clarifying policy understandings. Milbank Q.

[CR40] Grant WB (2009). How strong is the evidence that solar ultraviolet B and vitamin D reduce the risk of cancer? An examination using Hill’s criteria for causality. Dermatoendocrinology.

[CR41] Green LW, Kreuter MW (1999). Health promotion planning: an educational and ecological approach.

[CR42] Greenland S (2003). Quantifying biases in causal models: classical confounding vs collider-stratification bias. Epidemiology.

[CR43] Greenland S, Pearl J, Robins JM (1999). Causal diagrams for epidemiologic research. Epidemiology.

[CR44] Haddon W (1970). On the escape of tigers, an ecological note. Am J Public Health.

[CR45] Haddon W (1980). Advances in the epidemiology of injuries as a basis for public policy. Public Health Rep.

[CR46] Haddon W, Suchman E, Klein D (1964). Accident research: methods and approaches.

[CR47] Hagel B, Meeuwisse W (2004). Risk compensation: a “side effect” of sport injury prevention. Clin J Sport Med.

[CR48] Hanson D, Hanson J, Vardon P, McFarlane K, Lloyd J, Muller R (2005). The injury iceberg: an ecological approach to planning sustainable community safety interventions. Health Promot J Austr.

[CR49] Hanson DW, Finch CF, Allegrante JP, Sleet D (2012). Closing the gap between injury prevention research and community safety promotion practice: revisiting the public health model. Public Health Rep.

[CR50] Heinrich HW (1931). Industrial accident prevention: a scientific approach.

[CR51] Hernán MA (2015). Invited commentary: agent-based models for causal inference—reweighting data and theory in epidemiology. Am J Epidemiol.

[CR52] Hill AB (1965). The environment and disease: association or causation?. Proc R Soc Med.

[CR53] Huebner RJ (1957). Criteria for etiologic association of prevalent viruses with prevalent diseases; the virologist’s dilemma. Ann N Y Acad Sci.

[CR54] Ip EH, Rahmandad H, Shoham DA, Hammond R, Huang TK, Wang Y (2013). Reconciling statistical and systems science approaches to public health. Health Educ Behav.

[CR55] Jenkins DP, Salmon PM, Stanton NA, Walker GH (2010). A systemic approach to accident analysis: a case study of the Stockwell shooting. Ergonomics.

[CR56] Koopman JS, Lynch JW (1999). Individual causal models and population system models in epidemiology. Am J Public Health.

[CR57] Krieger N (1994). Epidemiology and the web of causation: has anyone seen the spider?. Soc Sci Med.

[CR58] Lalonde M. A new perspectice on the health of Canadians: a working document. http://www.phac-aspc.gc.ca/ph-sp/pube-pubf/perintrod-eng.php (1974). Accessed 24 Aug 2015.

[CR59] Leveson NG (2004). A new accident model for enginnering safer systems. Saf Sci.

[CR60] Leveson NG (2011). Applying systems thinking to analyze and learn from events. Saf Sci.

[CR61] Li G, Baker SP, Li G, Baker SB (2014). Preface. Injury research: theories, methods and approaches.

[CR62] Loomis D, Wing S (1990). Is molecular epidemiology a germ theory for the end of the twentieth century?. Int J Epidemiol.

[CR63] Luke DA, Stamatakis KA (2012). Systems science methods in public health. Annu Rev Public Health.

[CR64] Lyon A (1967). Causality. Br J Philos Sci.

[CR65] Mackie JL (1965). Causes and conditions. Am Philos Q.

[CR66] MacMahon B, Pugh TF, Ipsen J (1960). Epidemiologic methods.

[CR67] Marshall BDL, Galea S (2014). Formalizing the role of agent-based modeling in causal inference and epidemiology. Am J Epidemiol.

[CR68] Marshall BDL, Paczkowski, MN, Seemann L, Tempalski B, Pouget RR, et al. A complex systems approach to evaluate HIV prevention in metropolitan areas: preliminary implications for combination intervention strategies. Plos One. 2012; doi:10.1371/journal.pone.0044833. 10.1371/journal.pone.0044833 PMC3441492 23028637

[CR69] McGlashan AJ, Finch CF (2010). The extent to which behavioural and social sciences theories and models are used in sport injury prevention research. Sports Med.

[CR70] McIntosh A (2005). Risk compensation, motivation, injuries, and biomechanics in competitive sport. Br J Sports Med.

[CR71] McKeown T (1979). The role of medicine. Dream, mirage or nemesis?.

[CR72] Meeuwisse WH (1994). Assessing causation in sport injury: a multifactorial model. Clin J Sport Med.

[CR73] Meeuwisse WH (1994). Athletic injury etiology: distinguishing between interaction and confounding. Clin J Sport Med.

[CR74] Meeuwisse WH, Tyreman H, Hagel B, Emery C (2007). A dynamic model of etiology in sport injury: the recursive nature of risk and causation. Clin J Sport Med.

[CR75] Messier SP, Edwards DG, Martin DF, Lowery RB, Cannon DW, James MK (1995). Etiology of iliotibial band friction syndrome in distance runners. Med Sci Sports Exerc.

[CR76] Moore D, Dray A, Green R, Hudson SL, Jenkinson R, Siokou C (2009). Extending drug ethno-epidemiology using agent-based modelling. Addiction.

[CR77] Ness RB, Koopman JS, Robert MS (2007). Causal system modeling in chronic disease epidemiology: a proposal. Ann Epidemiol.

[CR78] Nielsen RO, Buist I, Sørensen H, Lind M, Rasmussen S (2012). Training errors and running related injuries: a systematic review. Int J Sports Phys Ther.

[CR79] Nielsen RO, Buist I, Parner ET, Nohr EA, Sorensen H, Lind M (2014). Foot pronation is not associated with increased injury risk in novice runners wearing a neutral shoe: a 1-year prospective cohort study. Br J Sports Med.

[CR80] Olobatuyi ME (2006). A user’s guide to path analysis.

[CR81] Parascandola M, Weed DL (2001). Causation in epidemiology. J Epidemiol Community Health.

[CR82] Pearce N, Merletti F (2006). Complexity, simplicity, and epidemiology. Int J Epidemiol.

[CR83] Pearl J (1995). Causal diagrams for empirical research. Biometrika.

[CR84] Phillips CV, Goodman KJ (2004). The missed lessons of Sir Austin Bradford Hill. Epidemiol Perspect Innov.

[CR85] Phillips CV, Goodman KJ (2006). Causal criteria and counterfactuals; nothing more (or less) than scientific common sense. Emerg Themes Epidemiol.

[CR86] Potischman N, Weed DL (1999). Causal criteria in nutritional epidemiology. Am J Clin Nutr.

[CR87] Rasmussen J (1997). Risk management in a dynamic society: a modelling problem. Saf Sci.

[CR88] Read G, Salmon PM, Lenne MG (2013). Sounding the warning bells: the need for a systems approach to rail level crossing safety. Appl Ergon.

[CR89] Reason J (1997). Managing the risks of organisational accidents.

[CR90] Resnicow K, Page SE (2008). Embracing chaos and complexity: a quantum change for public health. Am J Public Health.

[CR91] Rivara FP, Rivara FP, Cummings P, Koepsell TD, Grossman DC, Maier RV (2001). An overview of injury research. Injury control: a guide to research and evaluation.

[CR92] Rivers T (1937). Viruses and Koch’s postulates. J Bacteriol.

[CR93] Robertson LS (2007). Injury epidemiology.

[CR94] Rodrigues P, TenBroek T, Hamill J (2013). Runners with anterior knee pain use a greater percentage of their available pronation range of motion. J Appl Biomech.

[CR95] Rogers ES (1960). Human ecology and health: an introduction for administrators.

[CR96] Ronksley PE, Brien SE, Turner BJ, Mukamal KJ, Ghali WA (2011). Association of alcohol consumption with selected cardiovascular disease outcomes: a systematic review and meta-anlysis. Br Med J.

[CR97] Rose G (1985). Sick individuals and sick populations. Int J Epidemiol.

[CR98] Rothman KJ (1976). Causes. Am J Epidemiol.

[CR99] Rothman KJ, Greenland S (2005). Causation and causal inference in epidemiology. Am J Public Health.

[CR100] Runyan CW (1998). Using the Haddon matrix: introducing the third dimension. Inj Prev.

[CR101] Salmon PM, Williamson A, Lenné M, Mitsopoulos-Rubens E, Rudin-Brown CM (2010). Systems-based accident analysis in the led outdoor activity domain: application and evaluation of a risk management framework. Ergonomics.

[CR102] Salmon PM, Cornelissen M, Trotter MJ (2012). Systems-based accident analysis methods: a comparison of Accimap, HFACS, and STAMP. Saf Sci.

[CR103] Salmon PM, Goode N, Lenné MG, Finch CF, Cassell E (2014). Injury causation in the great outdoors: a systems analysis of led outdoor activity injury incidents. Accid Anal Prev.

[CR104] Saragiotto BT, Yamato TP, Hespanhol LC, Rainbow MJ, Davis IS, Lopes AD (2014). What are the main risk factors for running related injuries?. Sports Med.

[CR105] Scott-Parker B, Morang MacKay J (2015). Research and practice in a multidimensional world: a commentary on the contribution of the third dimension of the Haddon matrix to injury prevention. Inj Prev.

[CR106] Scott-Parker B, Goode N, Salmon P (2015). The driver, the road, the rules…and the rest? A systems-based approach to young driver road safety. Accid Anal Prev.

[CR107] Senge PM (1990). The fifth discipline: the art and practice of the learning organisation.

[CR108] Shipley B (2002). Cause and correlation in biology.

[CR109] Shrier I, Platt RW (2008). Reducing bias through directed acyclic graphs. BMC Med Res Methodol.

[CR110] Stapp JP (1957). Human tolerance to deceleration. Am J Surg.

[CR111] Sterman JD (2006). Learning from evidence in a complex world. Am J Public Health.

[CR112] Susser M (1998). Does risk factor epidemiology put epidemiology at risk? Peering into the future. J Epidemiol Community Health.

[CR113] Susser M, Susser E (1996). Choosing a future for epidemiology: I. Eras and paradigms. Am J Public Health.

[CR114] Susser M, Susser E (1996). Choosing a future for epidemiology: II. From black box to Chinese boxes and eco-epidemiology. Am J Public Health.

[CR115] Trifiletti LB, Gielen AC, Sleet DA, Hopkins K (2005). Behavioral and social sciences theories and models: are they used in unintentional injury prevention research?. Health Educ Res.

[CR116] Trochim WM, Cabera DA, Milstein B, Gallagher RS, Leischow SL (2006). Practical challenges of systems thinking and modeling in public health. Am J Public Health.

[CR117] van Gent RN, Siem D, van Middelkoop M, van Os AG, Bierma-Zeinstra SMA, Koes BW (2007). Incidence and determinants of lower extremity running injuries in long distance runners: a systematic review. Br J Sports Med.

[CR118] van Mechelen W, Hlobil H, Kemper HCG (1992). Incidence, severity, aetiology and prevention of sports injuries: a review of concepts. Sports Med.

[CR119] Vandenbroucke JP (1988). Is ‘The Causes of Cancer’ a miasma theory for the end of the twentieth century?. Int J Epidemiol.

[CR120] VanderWeele TJ, Robins JM (2007). Directed acyclic graphs, sufficient causes, and the properties of conditioning on a common effect. Am J Epidemiol.

[CR121] VanLeeuwen JA, Waltner-Toews D, Abernathy T, Smit B (1999). Evolving models of human health toward an ecosystem context. Ecosyst Health.

[CR122] Wing S (1984). The role of medicine in the decline of hypertension-related mortality. Int J Health Serv.

[CR123] Wing S (1988). Social inequalities in the decline of coronary mortality. Am J Public Health.

[CR124] Wing S (1994). The limits of epidemiology. Med Global Surv.

[CR125] World Health Organisation. The Ottawa Charter for health promotion: first international conference on health promotion, Ottawa, 21 November 1986. http://www.who.int/healthpromotion/conferences/previous/ottawa/en/. Accessed 23 Nov 2015.

[CR126] Yang Y, Diez Roux AV, Auchincloss AH, Rodriguez DA, Brown DG (2011). A spatial agent-based model for the simulation of adults’ daily walking within a city. Am J Prev Med.

